# Fecal Calprotectin in Combination With Standard Blood Tests in the Diagnosis of Inflammatory Bowel Disease in Children

**DOI:** 10.3389/fped.2020.609279

**Published:** 2021-03-05

**Authors:** Shaun S. C. Ho, Michael Ross, Jacqueline I. Keenan, Andrew S. Day

**Affiliations:** ^1^Department of Pediatrics, University of Otago, Christchurch, New Zealand; ^2^Department of Surgery, University of Otago, Christchurch, New Zealand

**Keywords:** inflammatory bowel disease, fecal calprotectin, serum albumin, platelet count, pediatrics, diagnosis

## Abstract

**Introduction:** Fecal calprotectin (FC) is a useful non-invasive screening test but elevated levels are not specific to inflammatory bowel disease (IBD). The study aimed to evaluate the sensitivity, specificity, positive predictive value (PPV) and negative predictive value (NPV) of FC alone or FC in combination with other standard blood tests in the diagnosis of IBD.

**Methods:** Children aged <17 years who had FC (normal range <50 μg/g) measured and underwent endoscopy over 33 months in Christchurch, New Zealand were identified retrospectively (consecutive sampling). Medical records were reviewed for patient final diagnoses.

**Results:** One hundred and two children were included; mean age was 12.3 years and 53 were male. Fifty-eight (57%) of the 102 children were diagnosed with IBD: 49 with Crohn's disease, eight with ulcerative colitis and one with IBD-unclassified. FC of 50 μg/g threshold provided a sensitivity of 96.6% [95% confident interval (CI) 88.3–99.4%] and PPV of 72.7% (95% CI 61.9–81.4%) in diagnosing IBD. Two children with IBD however were found to have FC <50 μg/g. Sensitivity in diagnosing IBD was further improved to 98.3% (95% CI 90.7–99.1%) when including FC >50 μg/g or elevated platelet count. Furthermore, PPVs in diagnosing IBD improved when FC at various thresholds was combined with either low albumin or high platelet count.

**Conclusion:** Although FC alone is a useful screening test for IBD, a normal FC alone does not exclude IBD. Extending FC to include albumin or platelet count may improve sensitivity, specificity, PPV and NPV in diagnosing IBD. However, prospective studies are required to validate this conclusion.

## Introduction

Inflammatory bowel disease (IBD) is a chronic inflammatory condition involving the gastrointestinal tract, encompassing Crohn's disease (CD), ulcerative colitis (UC) and IBD-unclassified (IBD-U). The incidence of IBD in New Zealand across all ages is known to be high by international standards, notably in the Canterbury region (1, 2). While the cause of IBD remains unclear, there is a growing awareness that a combination of host genetics and environmental factors that include diet and gut bacteria is likely to have a role in disease development (3).

In New Zealand, clinical guidelines produced by the National Pediatric Gastroenterology Clinical Network recommended blood (at least the following: full blood count, erythrocyte sedimentation rate (ESR), C-reactive protein (CRP) and albumin) and fecal tests to screen for the presence of systemic inflammation and exclude any gastrointestinal infections for any children suspected of having IBD (4). Measurement of fecal calprotectin (FC) is included as one of the initial investigations. Calprotectin is a 36-kDa heterodimer of the S100A8 and S100A9 proteins, which is released predominantly by neutrophils, and a lesser extent by monocytes and macrophages, in response to infection or inflammation (5, 6). Calprotectin found in feces is more specific to gastrointestinal inflammation than calprotectin found in other body fluids (7). Hence, FC has been found to be useful in screening purposes and distinguishing inflammatory from non-inflammatory gastrointestinal conditions such as irritable bowel syndrome (8).

The study hypothesized that combining FC with other standard blood tests improved the diagnosis of IBD compared to FC alone. This study aimed to explore various FC thresholds in the diagnosis of pediatric IBD, and to ascertain whether a combination of FC with other standard blood tests could improve the diagnostic utility.

## Methods

### Patient Selection

**Figure 1:**
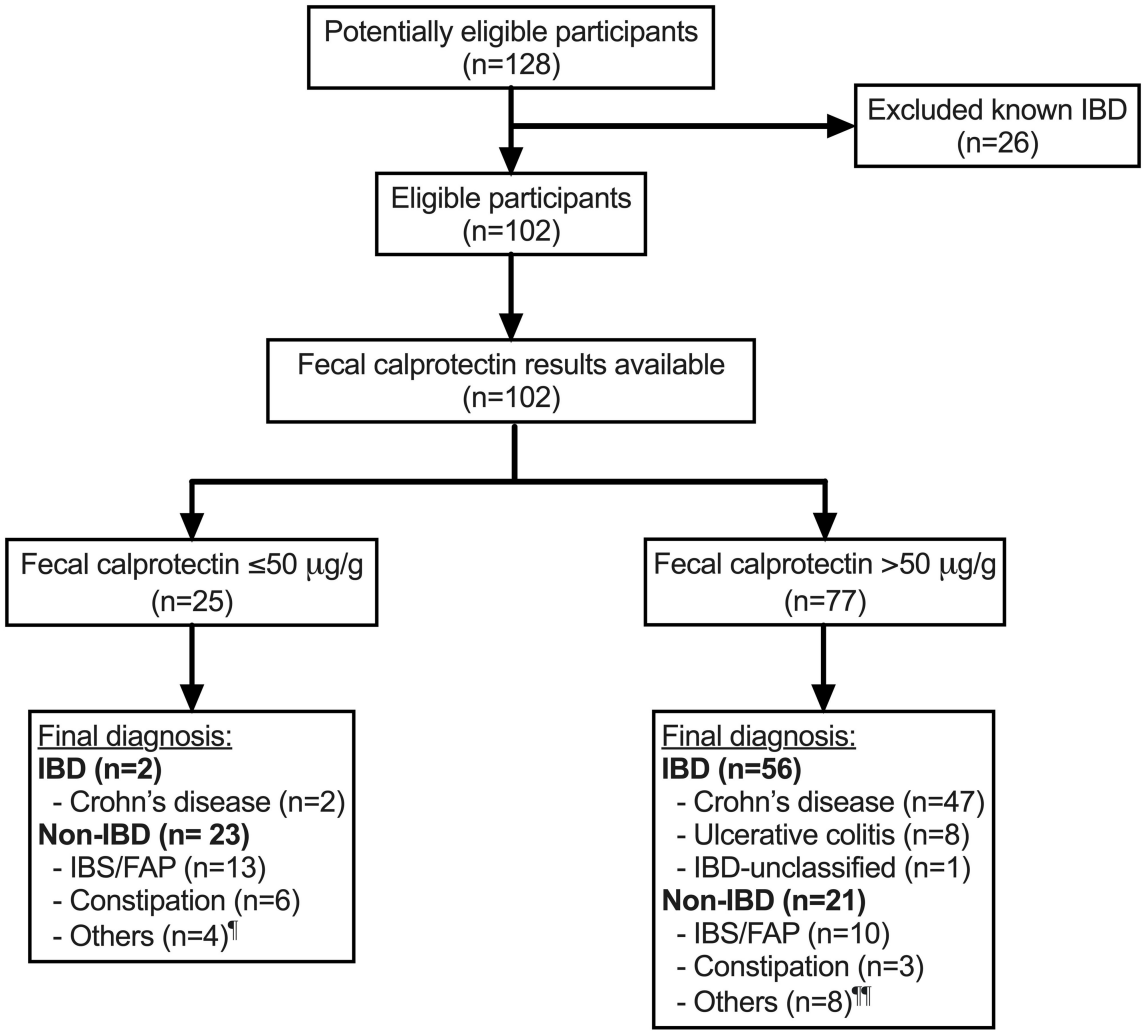
Flow of participants through the study. ^¶^ Others include *Helicobacter pylori* gastritis (*n* = 1), non-diagnostic following referral for hematochezia (*n* = 2) and iron deficiency (*n* = 1). ^¶¶^ Others include duodenitis of unknown etiology (*n* = 1), non-diagnostic following referral for hematochezia (*n* = 2), juvenile polyp (*n* = 1), protein losing enteropathy (*n* = 1), recurrent perianal abscess (*n* = 1), severe combined immune deficiency colitis (*n* = 1) and erythema nodosum (*n* = 1). IBD, inflammatory bowel disease; FAP, functional abdominal pain; IBS, irritable bowel syndrome; n, sample size.

Children and adolescents under 17 years of age who underwent colonoscopy at Christchurch Hospital, Canterbury, New Zealand between 1 January 2016 and 30 September 2018 (33 months) were retrospectively identified from the hospital database. The indications for endoscopy were either investigation of persistent symptoms, such as hematochezia, or to exclude IBD. The endoscopists were not blinded for the indication of investigation. Patients who had FC measured prior to their endoscopy investigation were included (Figure 1). These children were categorized into IBD or non-IBD groups. Diagnosis of IBD, inclusive of CD, UC and IBD-U, was made based on the European Society of Pediatric Gastroenterology, Hepatology and Nutrition (ESPGHAN) Revised Porto criteria (9) and the Paris classification (10).

Each patient's medical record was accessed to document the patient's background, results of any fecal, blood, imaging and histology investigations, disease extent in those diagnosed with IBD and final diagnosis for those with non-IBD. Exclusion criteria included children with known IBD, evidence of recent gastrointestinal infection or recent administration of non-steroid anti-inflammatory medications. Due to insufficient documentation of symptoms in the medical records, a symptoms variable was not included in the analysis.

This study was approved by the University of Otago Ethics Committee (Health). The full study protocol can be accessed by contacting the authors.

### Fecal Calprotectin Test

Measurement of FC levels was performed by a central local laboratory, Canterbury Health Laboratories (CHL) using Calpro Calprotectin ELISA assay (Calpro AS, Norway), usually with some clinical information on the request form. Such clinical information was not available and not included in the analysis of this study. The assay reported the normal range of FC as <50 μg/g and up to 500 μg/g. Increments of 50 μg/g were used to explore the optimal threshold (up to a maximum threshold of 250 μg/g) that best diagnosed IBD.

### Other Blood Tests

Blood results, including low albumin, elevated platelet count, erythrocyte sedimentation rate (ESR) or C-reactive protein (CRP) were likewise categorized as normal or abnormal based on the reference ranges provided by the two laboratories (CHL and Canterbury SCL) that serve the local region. (Normal reference ranges for CHL: albumin >1 year-8 years: 35–45 g/L, >8–15 years: 37–47 g/L and > 15 years: 32–48 g/L, platelet count: >1 year-3 years: 150–500 × 10^9^/L, >3–7 years: 150–475 × 10^9^/L, >7–12 years: 150–425 × 10^9^/L, >12 years: 150–400 × 10^9^/L, CRP: <5 mg/L, high sensitive CRP: <1.0 mg/L and ESR: 1–10 mm/h; Canterbury SCL: albumin 32–48 g/L, CRP: <5 mg/L, ESR: 1–10 mm/h, platelet count: >1 year-3 years: 150–500 × 10^9^/L, >3–7 years: 150–475 × 10^9^/L, >7–12 years: 150–425 × 10^9^/L, >12 years: 150–400 × 10^9^/L).

### Statistical Analysis

Statistical analysis was performed using GraphPad Prism version 9.0.0 (86) macOS, Version 26.0 (GraphPad Software, California, USA) for descriptive analysis, sensitivity (the proportion of children with IBD that the test correctly identifies as positive, true positive), specificity (the proportion of children with non-IBD that the test correctly identifies as negative, true negative), positive predictive value, PPV (the probability of a patient having IBD when the test is positive) and negative predictive value, NPV (the probability of a patient not having IBD when the test is negative). When PPV was reported to be the highest value in diagnosing IBD, odds ratio (OR) was also provided. Each value was accompanied by 95% confident interval (CI) calculated using the Wilson-Brown method (for sensitivity, specificity, PPV and NPV) and Baptista-Pike method (for OR). A two-tailed *p* < 0.05 was considered statistically significant.

Patients were identified by consecutive sampling based on the study period, which also determine the final sample size. Patients with missing data (e.g., blood results not available) were excluded from the analysis. In addition, results on missed IBD and colonoscopy not required were provided for each FC threshold alone or FC combined with standard blood results.

## Results

### Patient Backgrounds

One hundred and twenty-eight children were potentially eligible participants (Figure 1). After excluding 26 children with previously known IBD, 102 children with mean age (± standard deviation, SD) of 12.3 ± 3.5 years and 53 (52%) male were included in the analysis (Table 1). Of the 102 children, 58 (57%) were newly diagnosed with IBD: 49 with CD, eight with UC and one with IBD-U, and 44 (43%) did not have IBD (non-IBD). At least half of the children without IBD were diagnosed with functional abdominal pain or irritable bowel syndrome. A quarter of the whole cohort were found to have FC ≤50 μg/g (normal range) and the rest of the children had FC >50 μg/g. Overall, the mean (± SD) duration between measurement of FC and endoscopy was 2.0 ± 3.0 months.

**Table 1:** Background characteristics of 102 children and adolescents diagnosed with and without inflammatory bowel disease (IBD).

	**Total (*n* = 102)**	**IBD (*n* = 58)**	**Non-IBD (*n* = 44)**
**Age at diagnosis**, mean ± standard deviation	12.3 ± 3.5 years	12.3 ± 3.1 years	12.2 ± 4.0 years
**Male**, n (%)	53 (52)	33 (57)	20 (46)
**Ethnicity**, n			
New Zealand European/other European	99	56	43
Māori	6	2	4
Other Asian	3	1	2
Not specified	6	3	3
**IBD subtypes**, n (%)	N/A	58 (57)	N/A
Crohn's disease^†^		49 (85)	
Age at diagnosis			
0 to <10 years (A1a)		10	
10 to <17 years (A1b)		39	
Location			
Distal third of ileum ± cecal disease (L1)		6	
Colonic (L2)		12	
Ileocolonic (L3)		28	
Upper disease proximal to ligament of Trietz (L4a)		17	
Upper disease distal to ligament of Trietz (L4b)		2	
Upper disease to distal third of ileum (L4ab)		7	
Behavior			
Non-stricturing and non-penetrating (B1)		45	
Stricturing (B2)		2	
Penetrating (B3)		2	
Both structuring and penetrating disease (B2B3)		0	
Perianal disease (p)		11	
Ulcerative colitis^†^		8 (14)	
Extent			
Proctitis (E1)		0	
Left-sided colitis (E2)		2	
Extensive colitis (E3)		1	
Pancolitis (E4)		5	
Severity			
Never severe (S0)		8	
Ever severe (S1)		0	
IBD-unclassified		1 (2)	
**Non-IBD**, n (%)	N/A	N/A	44 (43)
Functional abdominal pain/irritable bowel syndrome			23
Constipation			9
Juvenile polyp			1
Others^‡^			11
**Patients with various fecal calprotectin thresholds**, n (%)			
≤ 50 μg/g^§^	25 (25)	2 (3)	23 (52)
≥50 μg/g	77 (76)	56 (97)	21 (48)
≥100 μg/g	72 (71)	54 (93)	18 (41)
≥150 μg/g	69 (68)	53 (91)	16 (36)
≥200 μg/g	66 (65)	51 (88)	15 (34)
≥250 μg/g	65 (64)	50 (86)	15 (34)
**Blood tests**, n (%)			
*Albumin*	93 (91)	54 (93)	39 (89)
Low albumin^††^	34 (37)	33 (61)	1 (3)
*Platelet count*	101 (99)	57 (56)	44 (44)
Elevated platelet count^††^	28 (28)	25 (44)	3 (7)
*Erythrocyte sedimentation rate (ESR)*	76 (75)	47 (62)	29 (38)
Elevated ESR^††^	41 (54)	36 (77)	5 (17)
*C-reactive protein (CRP)*	101 (99)	57 (56)	44 (44)
Elevated CRP^††^	41 (41)	34 (60)	7 (16)
**Duration between fecal calprotectin and endoscopy**, mean ± standard deviation	2.0 ± 3.0 months	0.9 ± 1.3 months	3.5 ± 3.8 months

§ *Standard laboratory threshold*.

† *Based on the Paris classification (10)*.

†† *Percentage was calculated based on the available test results*.

‡ *Others: iron deficiency (n = 1), Helicobacter pylori gastritis (n = 1), duodenitis of unknown etiology (n = 1), protein losing enteropathy (n = 1), severe combined immune deficiency colitis (n = 1), recurrent perianal abscess (n = 1), erythema nodosum (n = 1), and non-diagnostic following referral for hematochezia (n = 4)*.

*IBD, inflammatory bowel disease; N/A, not applicable*.

### Fecal Calprotectin (FC) Test

Using the standard lab reference (<50 μg/g), FC provided a sensitivity of 96.6% (95% CI 88.3–99.4%), specificity of 52.3% (95% CI 37.9–66.3%), PPV of 72.7% (95% CI 61.9–81.4%) and NPV of 92.0% (95% CI 75.0–98.6%) in diagnosing IBD (Table 2). Similarly, if the standard lab reference was applied as a screening test for the decision to undertake an endoscopy, 25 of 102 (25%) endoscopies would not have been required. However, two children with IBD would have been missed. One of these two children was an 8-year old child with CD involving the duodenal and ileocolonic regions, and the other was a 3-year old child with orofacial granulomatosis and perianal CD (non-luminal disease).

**Table 2:** Sensitivity, specificity, positive predictive values (PPV), negative predictive values (NPV), missed inflammatory bowel disease (IBD) diagnosis and number of endoscopies not required when various fecal calprotectin thresholds were used in diagnosing IBD.

**Fecal calprotectin threshold**	***n***	**Sensitivity, % (95% CI)**	**Specificity, % (95% CI)**	**PPV, % (95% CI)**	**NPV, % (95% CI)**	**Missed IBD, n (%)**	**Endoscopy not required, n (%)**
>50 μg/g	102	96.6 (88.3–99.4)	52.3 (37.9–66.3)	72.7 (61.9–81.4)	92.0 (75.0–98.6)	2 (2)	25 (25)
>100 μg/g	102	93.1 (83.6–97.3)	59.1 (44.4–72.3)	75.0 (63.9–83.6)	86.7 (70.3–94.7)	4 (4)	30 (29)
>150 μg/g	102	91.4 (81.4–96.3)	63.6 (48.9–76.2)	76.8 (65.6–84.2)	84.8 (69.1–93.4)	5 (5)	33 (32)
>200 μg/g	102	87.9 (77.1–94.0)	65.9 (51.1–78.1)	77.3^||^ (65.8–85.7)	80.6 (65.0–90.3)	7 (7)	36 (35)
>250 μg/g	102	86.2 (75.1–92.8)	65.9 (51.1–78.1)	76.9 (65.4–85.5)	78.4 (62.8–88.6)	8 (8)	37 (36)

|| *Odds ratio was 14.1 (95% CI 5.0–34.5), p < 0.0001 likelihood of having IBD*.

*CI, confident interval; n, sample size*.

Using various FC thresholds (50, 100, 150, 200, and 250 μg/g), a threshold of 200 μg/g was found to provide the highest PPV for the diagnosis of IBD (77.3%, 95% CI 65.8–85.7%) and OR 14.1 (95% CI 5.0–34.5, *p* < 0.0001) likelihood of having IBD (Table 2). In addition, the specificity of FC improved with higher thresholds, but did not improve beyond 200 μg/g. Similarly, a threshold of 200 μg/g was found to provide the highest PPV for the diagnosis of CD (73.7%, 95% CI 61.1–83.4%); these children were 11.6 times (95% CI 4.1–28.8, *p* < 0.0001) more likely to have IBD. The specificity of FC improved with higher thresholds, but not beyond 200 μg/g (maximum specificity of 65.9%, 95% CI 51.1–78.1%) (Table 3).

**Table 3:** Sensitivity, specificity, positive predictive values (PPV), negative predictive values (NPV), missed Crohn's disease (CD) diagnosis, and number of endoscopies not required when various fecal calprotectin thresholds were used in diagnosing CD.

**Fecal calprotectin threshold**	***n***	**Sensitivity, % (95% CI)**	**Specificity, % (95% CI)**	**PPV, % (95% CI)**	**NPV, % (95% CI)**	**Missed IBD, n (%)**	**Endoscopy not required, n (%)**
>50 μg/g	93	95.9 (86.3–99.3)	52.3 (37.9–66.3)	69.1 (57.4–78.8)	92.0 (75.0–98.6)	2 (2)	25 (27)
>100 μg/g	93	91.8 (80.8–98.8)	59.1 (44.4–72.3)	71.4 (59.3–81.1)	86.7 (80.3–94.7)	4 (4)	30 (32)
>150 μg/g	93	89.8 (78.2–95.6)	63.6 (48.9–76.2)	73.3 (61.0–72.9)	84.8 (69.1–93.4)	5 (5)	33 (35)
>200 μg/g	93	85.7 (73.3–92.9)	65.9 (51.1–78.1)	73.7^||^ (61.1–83.4)	80.6 (65.0–90.3)	7 (8)	36 (39)
>250 μg/g	93	83.7 (71.0–91.5)	65.9 (51.1–78.1)	73.2 (60.4–83.0)	78.4 (62.8–88.6)	8 (9)	37 (40)

|| *Odds ratio was 11.6 (95% CI 4.1–28.8), p < 0.0001 likelihood of having IBD*.

*CI, confident interval; n, sample size*.

### Fecal Calprotectin or Abnormal Blood Tests

Utilizing various FC thresholds or abnormal blood results (albumin, platelet count, ESR and CRP) improved the sensitivity and NPV in diagnosing IBD. Employing FC >50 μg/g or having an elevated platelet count increased sensitivity from 96.6% (95% CI 88.3–99.4%) to 98.3% (95% CI 90.7–99.1%) in diagnosing IBD, compared to FC >50 μg/g alone (Tables 2, 4). If these criteria were used for the endoscopy investigation decision, 23 of 102 (23%) endoscopies would not have been required and one patient with IBD would have been missed. The calculation of NPV in diagnosing IBD was found to be the highest at 96.0% when the FC threshold was set at 200 μg/g or low albumin was present (i.e., if one has FC <200 μg/g and normal albumin level, there is 96.0% (95% CI 80.5–99.8%) probability the child does not have IBD) (Supplementary Table 1).

**Table 4:** Sensitivity, specificity, positive predictive values (PPV), negative predictive values (NPV), missed inflammatory bowel disease (IBD) diagnosis and number of endoscopies not required when various fecal calprotectin thresholds or high platelet count were used in diagnosing IBD.

**Fecal calprotectin threshold (or high platelet count)**	***n***	**Sensitivity, % (95% CI)**	**Specificity, % (95% CI)**	**PPV, % (95% CI)**	**NPV, % (95% CI)**	**Missed IBD, n (%)**	**Endoscopy not required, n (%)**
>50 μg/g	101	98.3 (90.7–99.1)	50.0 (35.8–64.2)	71.8 (61.0–80.6)	95.7 (79.0–99.8)	1 (1)	23 (23)
>100 μg/g	101	96.5 (88.1–99.4)	54.5 (40.1–68.3)	73.3 (62.4–82.0)	92.3 (75.9–98.6)	2 (2)	26 (26)
>150 μg/g	101	96.5 (88.1–99.4)	56.8 (42.2–70.3)	74.7 (63.8–82.9)	92.6 (76.6–98.7)	2 (2)	27 (27)
>200 μg/g	101	94.7 (85.6–98.6)	59.1 (44.4–72.3)	75.0^||^ (63.9–83.6)	89.7 (73.6–96.4)	3 (3)	29 (29)
>250 μg/g	101	93.0 (83.3–97.2)	59.1 (44.4–72.3)	74.7 (63.5–83.3)	86.7 (70.3–94.7)	4 (4)	30 (30)

|| *Odds ratio was 26.0 (95% CI 7.1–85.9), p < 0.0001 likelihood of having IBD*.

*CI, confident interval; n, sample size*.

Sensitivity, specificity, PPV and NPV in diagnosing IBD were also analyzed using various FC cut-offs or having low albumin, elevated ESR and CRP (Supplementary Tables 1–3). None of these combinations improved the sensitivity or NPV better than a high platelet count except for low albumin, which was comparable. Furthermore, evaluating various FC thresholds or having either low albumin or high platelets did not improve sensitivity or NPV further in diagnosing IBD (Supplementary Table 4).

### Fecal Calprotectin and Abnormal Blood Tests

The specificity and PPV in diagnosing IBD could be further improved when FC of various thresholds was combined with either low albumin or high platelet count. Specificities and PPVs were 97.4% (95% CI 86.8–99.9%) and 97.3% (95% CI 86.2–99.9%) for FC >50 μg/g, 100% (95% CI 90.1–100%) and 100% (95% CI 90.1–100%) for FC >100, 150, 200, or 250 μg/g, respectively (Table 5).

**Table 5:** Sensitivity, specificity, positive predictive values (PPV), negative predictive values (NPV) when combined various fecal calprotectin thresholds with either low serum albumin or high platelet count were used in diagnosing IBD.

**Fecal calprotectin threshold and (low albumin or high platelet count)**	***n***	**Sensitivity, % (95% CI)**	**Specificity, % (95% CI)**	**PPV, % (95% CI)**	**NPV, % (95% CI)**
>50 μg/g	92	67.9 (54.5–78.9)	97.4 (86.8–99.9)	97.3 (86.2–99.9)	69.1 (56.0–79.7)
>100 μg/g	92	66.0 (52.6–77.3)	100 (91.0–100)	100^||^ (90.1–100)	68.4 (55.5–79.0)
>150 μg/g	92	64.2 (50.7–75.7)	100 (91.0–100)	100 (89.9–100)	67.2 (54.4–77.9)
>200 μg/g	92	60.4 (46.9–72.4)	100 (91.0–100)	100 (89.3–100)	65.0 (52.4–75.8)
>250 μg/g	92	60.4 (46.9–72.4)	100 (91.0–100)	100 (89.3–100)	65.2 (52.4–75.8)

|| *Odds ratio was infinity (95% CI 18.4-infinity), p < 0.0001 likelihood of having IBD*.

*CI, confident interval; n, sample size*.

## Discussion

The current study found the FC standard lab reference (<50 μg/g) provided an excellent sensitivity of 96.6% (95% CI 88.3–99.4%) but poor specificity (52.3%, 95% CI 37.9–66.3%) in diagnosing IBD in this group of children. Two children with IBD in this group were found to have normal FC levels. In children who had FC >50 μg/g, there was 72.7% (95% CI 61.9–81.4%) probability of being diagnosed with IBD (PPV). PPV marginally improved to 77.3% (95% CI 65.8–85.7%) if the FC threshold was increased to 200 μg/g. In addition, sensitivity in diagnosing IBD could be improved further (increasing from 96.6 to 98.3%) if the criteria included either a FC threshold of >50 μg/g or an elevated platelet count. On the other hand, the combination of a FC threshold >50 μg/g with either low serum albumin or high platelet count increased PPV in diagnosing IBD from 72.7 to 97.3% and 100% if FC >100 μg/g threshold was used.

FC as a stand-alone test is a highly sensitive but not specific test in identifying pediatric IBD. Systemic review and meta-analysis studies have reported FC sensitivity ranging from 0.92 to 0.99 and specificity from 0.65 to 0.76 in the utility of diagnosing pediatric IBD (11–14). The sensitivity of FC (using the standard lab threshold) reported in the current study is consistent (96.6%) with the current literature but marginally lower specificity (52.3%). Furthermore, FC is found to be a far superior test than standard blood tests (albumin, ESR, CRP, hemoglobin, white cell count or platelet count) in diagnosing IBD among children suspected of having IBD (15).

A number of studies have evaluated the usefulness of FC in the primary-care setting where IBD can be safely excluded and avoid unnecessary referrals or endoscopy (16–18). The most recent study by Walker et al. (18) demonstrated that a FC >100 μg/g is able to fully discriminate (100% sensitivity) children with IBD from those who do not have IBD in a primary-care setting. Nevertheless, in the present study (in a tertiary hospital setting) two children with IBD had normal FC results (<50 μg/g). One was an 8-year old child diagnosed with CD involving the duodenal and ileocolonic regions (non-stricturing). This child also found to have concurrent elevated inflammatory markers (platelet count, ESR and CRP). The other case was a 3-year old child with orofacial granulomatosis and perianal CD without any luminal involvement when FC was measured. This child also had normal inflammatory markers as part of the initial workup. However, when the child subsequently developed colonic disease, the FC was noted to be elevated. Two earlier studies also reported a small number of children with normal FC levels at the time of diagnosis of IBD (19, 20); the ESPGHAN GROWTH CD study (20) reported normal FC values in three of 60 children with newly diagnosed CD and a Scottish study showed this in two of 46 children with IBD (19). Hence, the clinical decision to refer to a gastroenterologist or proceed with endoscopy should not solely rely on FC measurement alone, but should take consideration of the clinical features and results of other available investigations.

Although it is uncommon for children with IBD to have a normal FC, inflammatory markers such as ESR or CRP are commonly elevated. The study by Quail et al. (19) reported two of 46 children with IBD had normal FC, but all children had at least one abnormal blood test and/or elevated FC at diagnosis. The current study analyzed the diagnostic sensitivity in IBD (avoiding missing true positive) using either FC (various thresholds) or abnormal inflammatory markers (low albumin, elevated platelets, CRP or ESR). When FC level was elevated (≥50 μg/g) or platelet count elevated, sensitivity in the diagnosis of IBD increased from 96.6 to 98.3%. If such criteria (FC ≥50 μg/g or elevated platelet count) were applied for endoscopy investigation decision, 23 children would not require an endoscopy assessment with one child with IBD (a child with non-luminal CD involvement). Interpretation of this analysis may potentially be biased toward CD as the majority (85%) of children in the current cohort were diagnosed with CD. A recent prospective study evaluated four diagnostic strategies (symptoms alone, symptoms plus blood markers (elevated CRP or anemia), symptoms plus FC (≥250 μg/g) and symptoms plus blood markers plus FC) to predict IBD diagnosis in 193 children aged between 6 and 18 years with persistent non-bloody diarrhea and abdominal pain (21). This study found the combination of symptoms with blood and stool markers was the best strategy to predict IBD in these children, with a superior area under the curve (AUC) of 0.997 compared to the other strategies (21). Nevertheless, this strategy is not applicable to children presenting with rectal bleeding or perianal disease.

Similarly, improving IBD diagnostic accuracy by combining FC with symptoms suggestive of IBD has also been evaluated (22, 23). A meta-analysis study of eight studies (*n* = 1,120) found FC to add the most diagnostic value to symptoms by reducing the children with IBD incorrectly classified as low risk of IBD from 16 to 9% (22). While in the primary care setting, a prospective study involving 90 children with chronic gastrointestinal symptoms found the most optimal strategy to identify patients of being high risk for IBD was to add FC to alarm symptoms (such as hematochezia, first-degree family history of IBD, involuntary weight loss, growth failure, extra-intestinal features or perianal lesions), which significantly increased the AUC compared to alarm symptoms alone (0.80 and 0.97, respectively) (23). The retrospective nature of the current study limited the ability to ascertain the presenting symptoms for all children: consequently, this was not able to be evaluated.

In most locations, access to endoscopy is limited and clinicians are required to prioritize patients accordingly. In order to assist such prediction (establishing which patients truly have IBD), the current study and two other reports have evaluated FC results in combination with various tests (24, 25). All studies found combining FC with another test to be superior to FC alone. The current study found that combining FC (>50 μg/g) with either low serum albumin or high platelet count increased PPV in diagnosing IBD from 72.7 to 97.3%, compared to FC alone. This suggests that patients with such test results should be prioritized for endoscopy. The study by Daniluk et al. (24) evaluated 128 children with chronic gastrointestinal symptoms with FC >150 μg/g and found that FC combined with ESR, CRP or albumin provided the best PPV (82.1% and AUC 0.92; *P* = 0.04) in diagnosing IBD. Similarly, another study that evaluated 77 children with chronic gastrointestinal symptoms found FC (>200 μg/g) and intestinal ultrasound (US) gave PPV of 100% in diagnosing IBD (25). All these studies were analyzed retrospectively and prospective studies are required to validate this further.

The current study was limited by the retrospective study design, meaning that the medical team, pathologists or patients were not blinded and that not all subjects had a complete series of blood tests. There was a variable period of time between the FC testing and the endoscopy assessment consequent to endoscopy waiting times. It would be ideal for the interval between FC testing and endoscopy assessment to be as short as possible, allowing for FC results to more closely reflect the mucosal pathology. The FC results analyzed by incremental thresholds were treated as a dichotomous variable as the laboratory did not perform further dilution for any results beyond 500 μg/g, which limits the calculation of optimal FC threshold using receiver operating characteristic curve analysis that would best distinguish IBD from non-IBD in this cohort of patients. Finally, this was a relatively small sample size with most of the children diagnosed with IBD classified as CD, which may have influenced the utility of the tests. However, each of the children was seen and investigated within the same pediatric unit.

In conclusion, FC alone is a useful screening test for IBD. However, a normal FC alone does not exclude IBD. The approach to combine FC with serum albumin or platelet count may improve sensitivity, specificity, PPV and NPV in diagnosing IBD. Furthermore, prospective studies are required to validate this conclusion.

## Data Availability Statement

The raw data supporting the conclusions of this article will be made available by the authors, without undue reservation.

## Ethics Statement

The studies involving human participants were reviewed and approved by The University of Otago Ethics Committee (Health). Written informed consent for participation was not provided by the participants' legal guardians/next of kin because: This is a retrospective study and has no contact with human subjects. All data obtained from hospital or lab records.

## Author Contributions

SSCH was involved in study design, data entry, analyzed and interpreted the data, and wrote the initial draft of the manuscript. MR was involved in study design and data entry. JK was involved in study conception and supervision. AD was involved in study conception and design, analysis and interpretation of data arising, and supervision. All authors were involved in the critical review of the manuscript and all have approved and are accountable for the final version of the manuscript.

## Conflict of Interest

The authors declare that the research was conducted in the absence of any commercial or financial relationships that could be construed as a potential conflict of interest.
