# Supplementary material for: Fecal Calprotectin in Combination With Standard Blood Tests in the Diagnosis of Inflammatory Bowel Disease in Children
Source: Front Pediatr. 2021 Mar 5;8:609279. doi: 10.3389/fped.2020.609279 (PMC7973106; doi:10.3389/fped.2020.609279)
Supplement: Supplementary file 1 [file Table_1.docx]

# SUPPLEMENTARY TABLES:

**Supplementary Table 1: Sensitivity, specificity, positive predictive values (PPV), negative predictive values (NPV), missed inflammatory bowel disease (IBD) diagnosis and number of endoscopies not required when various fecal calprotectin thresholds or low serum albumin were used in diagnosing IBD.**

| **Fecal calprotectin threshold (or low albumin)** | **n** | **Sensitivity, % (95% CI)** | **Specificity, % (95% CI)** | **PPV, % (95% CI)** | **NPV, % (95% CI)** | **Missed IBD, n (%)** | **Endoscopy not required, n (%)** |
| --- | --- | --- | --- | --- | --- | --- | --- |
| >50µg/g | 93 | 98.2  (90.2-99.9) | 48.7  (33.9-63.8) | 72.6  (61.4-81.5) | 95.0  (76.4-99.7) | 1 (1) | 20 (22) |
| >100µg/g | 93 | 98.2  (90.2-99.9) | 56.4  (41.0-70.7) | 75.7  (64.5-84.3) | 95.7  (79.0-99.8) | 1 (1) | 23 (25) |
| >150µg/g | 93 | 98.2  (90.2-99.9) | 59.0  (43.4-72.9) | 76.8  (65.6-85.2) | 95.8  (79.8-99.8) | 1 (1) | 24 (26) |
| >200µg/g | 93 | 98.2  (90.2-99.9) | 61.5  (45.9-75.1) | 77.9^\|\|^  (66.7-86.2) | 96.0  (80.5-99.8) | 1 (1) | 25 (27) |
| >250µg/g | 93 | 96.3  (87.5-99.3) | 61.5  (45.9-75.1) | 77.6  (66.3-85.9) | 92.3  (75.9-98.6) | 2 (2) | 26 (28) |

^||^ Odds ratio was 84.8 (95% CI 12.2-888.6), p<0.0001 likelihood of having IBD.

CI, confident interval; n, sample size.

**Supplementary Table 2: Sensitivity, specificity, positive predictive values (PPV), negative predictive values (NPV), missed inflammatory bowel disease (IBD) diagnosis and number of endoscopies not required when various fecal calprotectin thresholds or elevated erythrocyte sedimentation rate (ESR) were used in diagnosing IBD.**

| **Fecal calprotectin threshold (or elevated ESR)** | **n** | **Sensitivity, % (95% CI)** | **Specificity, % (95% CI)** | **PPV, % (95% CI)** | **NPV, % (95% CI)** | **Missed IBD, n (%)** | **Endoscopy not required, n (%)** |
| --- | --- | --- | --- | --- | --- | --- | --- |
| >50µg/g | 76 | 97.9  (88.9-99.9) | 44.8  (28.4-62.5) | 74.2  (62.1-83.5) | 92.9  68.5-99.6) | 1 (1) | 14 (18) |
| >100µg/g | 76 | 95.7  (85.8-99.2) | 48.3  (31.4-64.6) | 75.0  (62.8-84.2) | 87.5  (64.0-97.8) | 2 (3) | 16 (21) |
| >150µg/g | 76 | 95.7  (85.8-99.2) | 51.7  (34.4-68.6) | 76.3  (64.0-85.3) | 88.2  (65.7-97.9) | 2 (3) | 17 (22) |
| >200µg/g | 76 | 93.6  (82.8-97.8) | 55.2  (37.6-71.6) | 77.2^\|\|^  (64.8-86.2) | 84.2  (62.4-94.5) | 3 (4) | 19 (25) |
| >250µg/g | 76 | 93.6  (82.8-97.8) | 55.2  (37.5-71.6) | 77.2^\|\|^  (64.8-86.2) | 84.2  (62.4-94.5) | 3 (4) | 19 (25) |

^||^ Odds ratio was 18.1 (95% CI 4.7-62.5), p<0.0001 likelihood of having IBD.

CI, confident interval; n, sample size.

**Supplementary Table 3: Sensitivity, specificity, positive predictive values (PPV), negative predictive values (NPV), missed inflammatory bowel disease (IBD) diagnosis and number of endoscopies not required when various fecal calprotectin thresholds or elevated C-reactive protein (CRP) were used in diagnosing IBD.**

| **Fecal calprotectin threshold (or elevated CRP)** | **n** | **Sensitivity, % (95% CI)** | **Specificity, % (95% CI)** | **PPV, % (95% CI)** | **NPV, % (95% CI)** | **Missed IBD, n (%)** | **Endoscopy not required, n (%)** |
| --- | --- | --- | --- | --- | --- | --- | --- |
| >50µg/g | 101 | 98.3  (90.7-99.9) | 43.2  (29.7-57.8) | 69.1  (58.4-78.4) | 95.0  (76.4-99.7) | 1 | 20 |
| >100µg/g | 101 | 96.5  (88.1-99.4) | 47.7  (33.8-62.1) | 70.5  (59.6-79.5) | 91.3  (73.2-98.5) | 2 | 23 |
| >150µg/g | 101 | 96.5  (88.1-99.4) | 50.0  (35.8-64.1) | 71.4  (60.5-80.3) | 91.7  (74.2-98.5) | 2 | 24 |
| >200µg/g | 101 | 94.7  (85.6-98.6) | 52.3  (37.9-66.3) | 72.4^\|\|^  (61.4-80.9) | 88.5  (71.0-96.0) | 3 | 26 |
| >250µg/g | 101 | 93.0  (83.3-97.2) | 52.3  (37.9-66.3) | 71.6  (60.5-80.6) | 85.2  (67.5-94.1) | 4 | 27 |

^||^ Odds ratio was 19.7 (95% CI 5.4-65.4), p<0.0001 likelihood of having IBD.

CI, confident interval; n, sample size.

**Supplementary Table 4: Sensitivity, specificity, positive predictive values (PPV), negative predictive values (NPV), missed inflammatory bowel disease (IBD) diagnosis and number of endoscopies not required when various fecal calprotectin thresholds or low serum albumin or high platelet count were used in diagnosing IBD.**

| **Fecal calprotectin threshold (or low albumin or high platelet count)** | **n** | **Sensitivity, % (95% CI)** | **Specificity, % (95% CI)** | **PPV, % (95% CI)** | **NPV, % (95% CI)** | **Missed IBD, n (%)** | **Endoscopy not required, n (%)** |
| --- | --- | --- | --- | --- | --- | --- | --- |
| >50µg/g | 92 | 98.1  (90.1-99.9) | 46.2  (31.6-61.4) | 71.2  (60.0-80.4) | 94.7  (75.4-99.7) | 1 (1) | 19 (21) |
| >100µg/g | 92 | 98.1  (90.1-99.9) | 51.3  (36.2-66.1) | 73.2  (62.0-82.2) | 95.2  (77.3-99.8) | 1 (1) | 21 (23) |
| >150µg/g | 92 | 98.1  (90.1-99.9) | 53.9  (38.6-68.4) | 74.3  (63.0-83.1) | 95.5  (78.2-99.8) | 1 (1) | 22 (24) |
| >200µg/g | 92 | 98.1  (90.1-99.9) | 56.4  (41.0-70.7) | 75.4^\|\|^  (64.0-84.0) | 95.7  (79.0-99.8) | 1 (1) | 23 (25) |
| >250µg/g | 92 | 96.2  (87.3-99.3) | 56.47  (41.0-70.7) | 75.0  (63.6-83.8) | 91.7  (74.2-98.5) | 2(2) | 24 (26) |

^||^ Odds ratio was 67.3 (95% CI 9.7-708.1), p<0.0001 likelihood of having IBD.

CI, confident interval; n, sample size.
